# Genomic and Transcriptional Analysis of the Necroptosis Pathway Elements RIPK and MLKL in Sea Cucumber, *Holothuria leucospilota*

**DOI:** 10.3390/genes15101297

**Published:** 2024-10-03

**Authors:** Rong Chen, Qianying Huang, Yingzhu Rao, Junyan Wang, Ruiming Yu, Shuangxin Peng, Kaiyi Huang, Yihang Huang, Xiangxing Zhu, Dongsheng Tang, Xiaoli Zhang, Tiehao Lin, Ting Chen, Aifen Yan

**Affiliations:** 1 Mangrove Rare and Endangered Species Protection and Utilization Engineering Technology Research Center, Institute of Applied Biotechnology, School of Life Science and Technolog, Lingnan Normal University, Zhanjiang 528048, China; zsuchenrong@163.com (R.C.); raoyz@lingnan.edu.cn (Y.R.); 2 School of Medicine, Foshan University, Foshan 528000, China; hqy1802485136@163.com (Q.H.); 18938181981@163.com (J.W.); 13508294875@163.com (S.P.); a15723565471@outlook.com (K.H.); 13539371352@163.com (Y.H.); zhu_xiangxing@126.com (X.Z.); tangdsh@163.com (D.T.); zhangxiaoli0105@163.com (X.Z.); 3 School of Global Public Health, New York University, New York, NY 10012, USA; ry2143@nyu.edu; 4 Guangdong Institute for Drug Control, Guangzhou 5106630, China; thlin@tom.com; 5 Key Laboratory of Breeding Biotechnology and Sustainable Aquaculture (CAS), Key Laboratory of Tropical Marine Bio-Resources and Ecology, South China Sea Institute of Oceanology, Chinese Academy of Sciences, Guangzhou 510301, China; chan1010@scsio.ac.cn; 6 Research Centre on Aquaculture Nutrition and Environmental Ecology of the Ministry of Agriculture and Rural Affair, Shanghai Ocean University, Shanghai 201306, China

**Keywords:** *Holothuria leucospilota*, echinoderm, necroptosis, pathogenic or environmental challenges

## Abstract

**Background:** Receptor-interacting protein kinases (RIPKs) and mixed-lineage kinase domain-like protein (MLKL) are crucial in regulating innate immune responses and cell death signaling (necroptosis and apoptosis), and are potential candidates for genetic improvement in breeding programs. Knowledge about the RIPK family and MLKL in sea cucumber remains limited. **Methods:** We searched the genomes of sea cucumber *Holothuria leucospilota* for genes encoding RIPKs and MLKL, performed phylogenetic tree, motif and functional domain analyses, and examined tissue distribution and embryonic development patterns using qPCR. **Results:** RIPK5 (*Hl*-RIPK5), RIPK7 (*Hl*-RIPK7) and MLKL (*Hl*-MLKL) were identified in sea cucumber *H*. *leucospilota*. *Hl*-RIPK5 and *Hl*-RIPK7 were mainly expressed in coelomocytes, suggesting that they play a role in innate immunity, whereas *Hl*-MLKL exhibited relatively low expression across tissues. During embryonic development, *Hl*-MLKL was highly expressed from the 2-cell stage to the morula stage, while *Hl*-RIPK5 and *Hl*-RIPK7 were primarily expressed after the morula stage, indicating different roles in embryonic development. In primary coelomocytes, *Hl*-RIPK5 transcriptional activity was significantly depressed by LPS, poly(I:C), or pathogen *Vibrio harveyi*. *Hl*-RIPK7 expression levels were unchanged following the same challenges. *Hl*-MLKL mRNA levels were significantly decreased with poly(I:C) or *V. harveyi*, but did not change with LPS. **Conclusions:** These findings provide valuable insights into the evolutionary tree and characterization of RIPK and MLKL genes in sea cucumber, contributing to the broader understanding of the RIPK gene family and MLKL in ancient echinoderms.

## 1. Introduction

Receptor-interacting protein kinases (RIPKs), short for receptor-interacting protein kinases, are a family of Ser/Thr kinases that play crucial roles in both innate immunity and necroptosis, while mixed-lineage kinase domain-like protein (MLKL) is the key executor of necroptosis [1,2,3]. Previous research has shown that the RIPK1–RIPK3 complex activates RIPK3, which phosphorylates MLKL, leading to membrane disruption and triggering necroptosis [3,4,5].

Recent studies also suggest that the RIPK family shows atypical lives of typical kinase [6]. The RIPK family contains seven members, RIPK1–RIPK7, and all have a conserved kinase domain and distinct non-kinase domains [3]. RIPK1–RIPK4 have a conserved N-terminal kinase domain, while RIPK5–RIPK7 have a conserved C-terminal kinase domain. RIPK1 has a C-terminal death domain (DD) and a RIP homotypic interaction motif (RHIM) domain, and was considered as the founding member of the RIPK family [7]. RIPK2 has a C-terminal caspase-activation-and-recruitment domain (CARD). RIPK3 also has an RHIM domain but lacks a DD domain. The RHIM domain is important for mediating the assembly of a RIPK1 and RIPK3 complex termed the necrosome [8]. RIPK4 has C-terminal ankyrin repeats. RIPK5 contains a C-terminal kinase domain and a large unconserved N-terminal domain [9]. RIPK6 (LRRK1) and RIPK7 (LRRK2) have a leucine-rich repeat (LRR) domain, a ankyrin repeat (Ank) domain, Ras (GTPase) of complex proteins (ROC), and a carboxyl terminus of ROC (COR) domains.

Current research indicates that RIPK1–RIPK3 kinases contribute to distinct, but highly related innate immune response [3]; however, the studies of other RIPKs are still limited. RIPK1 interacts with RIPK3 to form an insoluble amyloid-like RIPK1 and RIPK3 necrosome complex, then activates a downstream pseudokinase, the mixed-lineage kinase domain-like protein (MLKL), and eventually induces necroptosis [10,11]. MLKL contains a C-terminal pseudokinase domain, a two-helix brace or linker, and an N-terminal four-helix bundle (4HB) domain. The 4HB domain is actually the executive domain performing the function of membrane permeabilization [12]. MLKL is well known for its functional role in necroptosis. In addition to this, MLKL also has non-necroptotic functions such as limiting intracellular bacteria replication and promoting nerve regeneration [13]. In addition to necroptosis, RIPK1 can also function as a key regulator of apoptosis and inflammation [14]. The main function of RIPK2 and RIPK4 is to activate NF-κB [6]. The biological function of RIPK5 remains poorly understood. Recent studies have linked RIPK6 and RIPK7 to the pathogenesis of Parkinson’s disease [15,16].

*Holothuria leucospilota* is a dominant tropical sea cucumber species naturally distributed in the Indo-Pacific region with high economic and ecological value [17]. The successful genome sequencing of *H. leucospilota* may provide basics for developing genome-wide studies in this species [18,19,20,21]. As an ancient species, sea cucumbers lack an adaptive immune system and rely overwhelmingly on innate immunity to defend against pathogens. Innate immunity is an evolutionarily ancient form of host defense against pathogens [22]. Upon detecting an invading pathogen, pattern recognition receptors (PRRs) rapidly and robustly transduce signaling cascades, allowing for effective antimicrobial responses, including the production of antimicrobial peptides, cytokines, and chemokines, or the induction of cell death, thus enabling effective control of infection [23]. The RIPK family, particularly the RIPK1–RIPK3–MLKL pathway, is considered to be a key decision-maker in innate immunity, especially in the induction of necroptosis [24]. The members of the RIPK family are ubiquitous and highly conserved in vertebrates [25]. The RIPK3 gene only appears in vertebrates and is presently unidentified in Cephalochordata and Urochordata [26]. Previous studies reported that the RIPK1 gene was found in echinoderms and phylum Hemichordata, as well as the Cephalochordate subphylum and Urochordata subphylum; however, these RIPK1 genes found in these species lack the conserved DD domain as their counterpart in vertebrates, and whether these genes are truly RIPK1 remains to be further investigated.

Hence, leveraging the whole-genome sequences [17] and the transcriptome data [27], a comprehensive genome-wide analysis was conducted to investigate the RIPK family and MLKL in the sea cucumber (*H. leucospilota*). The mRNA expression profiles of these genes across various adult tissues and developmental stages were assessed using quantitative PCR. Additionally, the transcriptional response of the RIPK5, RIPK7 and MLKL to the pathogenic or environmental challenges in the cultured primary coelomocytes was analyzed. This study provides valuable resources on the sea cucumber RIPK family and MLKL, which will enhance our understanding of their roles in host defense functions.

## 2. Materials and Methods

### 2.1. Cross-Genomic Analysis of the RIPK Gene Family and the MLKL Gene

To identify the genes of the RIPK family and the MLKL gene, nine representative deuterostome species were selected from the NCBI database for cross-genomic analysis compared with that of sea cucumber *H. leucospilota* (GenBank: PRJNA747844) [17]. The species utilized for analysis include *Homo sapiens* (GCA_000001405.29), *Gallus gallus* (GCA_016699485.2), *Xenopus tropicalis* (GCA_000004195.4), *Danio rerio* (GCA_000002035.6), *Ciona intestinalis* (GCA_000224145.3), *Branchiostoma lanceolatum* (GCA_900088365.1), *Saccoglossus kowalevskii* (GCF_000003605.2), *Acanthaster planci* (GCF_001949145.1), and *Apostichopus japonicas* (GCA_002754855.1).

### 2.2. Molecular Cloning of Hl-RIPK5, Hl-RIPK7 and Hl-MLKL ORF cDNA

RT-PCR was performed to clone the three analyzed genes, *Hl*-RIPK5, *Hl*-RIPK7, and *Hl*-MLKL, from sea cucumber *H. leucospilota*. Total RNA was extracted from the coelomocytes of *H. leucospilota* according to the normal protocol of TRIzol reagent (Invitrogen, Carlsbad, CA, USA) and subsequently reverse-transcribed to synthesize first-strand cDNA with the PrimeScript™ II 1st Strand cDNA Synthesis Kit (Takara, Kusatsu, Japan). To obtain and confirm the ORF sequences, PCR was performed with gene-specific primers (Table S1), which were designed based on the transcriptomic library of *H. leucospilota* coelomocytes previously constructed by our lab.

### 2.3. Phylogenetic Tree, Motif, and Structural Domain Analysis

The phylogenetic trees of the RIPK family and MLKL from 10 species (Table S2) were constructed using the neighbor-joining method (pairwise deletion) with 1000 bootstrap replicates in MEGA X software v10.0.5. The online databases including the InterPro v5.27-66.0 (https://www.ebi.ac.uk/interpro/, accessed on 1 September 2022), SMART v9.0 (https://smart.embl.de/), and CD-search v3.21 (https://www.ncbi.nlm.nih.gov/Structure/cdd/wrpsb.cgi/, accessed on 1 September 2022) were used to analyze the functional domains of the RIPK family and MLKL. The deduced protein sequences of *Hl*-RIPK5, *Hl*-RIPK7, and *Hl*-MLKL from sea cucumber *H. leucospilota* were aligned with corresponding sequences from other analyzed species (Table S2) to form three distinct groups, which were then submitted to MEME (Multiple Expectation Maximization for Motif Elicitation, https://meme-suite.org, accessed on 1 September 2022) for the identification of conserved motifs. The amino acid numbers of expected motifs were limited from 6 to 100.

### 2.4. Tissue Distribution and Ontogeny of Hl-RIPK5, Hl-RIPK7 and Hl-MLKL mRNA Expression

The tissue distribution of *Hl*-RIPK5, *Hl*-RIPK7, and *Hl*-MLKL mRNA was quantitatively detected in three individuals independently, with samples collected from a variety of tissues including body wall, muscle, oral tentacles, Cuvierian organ, coelomocytes, intestine, respiratory tree, Polian vesicles, transverse vessel, rete mirabile, ovaries, and testes. Additionally, embryos and larvae of the sea cucumber (*H. leucospilota*) were sampled at various developmental stages, including fertilized egg, 2 cells, 4 cells, 8 cells, 16 cells, morula, blastula, rotated-blastula, early-gastrula, late-gastrula, early-auricularia, mid-auricularia, auricularia, doliolaria, pentactula, and juvenile stages, as previously described [28]. The test samples of each developmental stages consisted of three groups, with each group comprising 30 pooled embryos.

Total RNA was extracted using TRIzol reagent, treated with gDNA Eraser (Takara, Kusatsu, Japan) to remove genomic DNA, and then reverse-transcribed with the PrimeScript™ RT Reagent Kit (Takara, Kusatsu, Japan) for quantitative PCR (qPCR). Specific primers (Table S1) were designed based on the obtained *Hl*-RIPK5, *Hl*-RIPK7 and *Hl*-MLKL cDNA sequences. qPCRs were performed using SYBR Premix ExTaq™ II (Takara, Kusatsu, Japan) in a final volume of 20 µL, with the conditions of 40 cycles of 95 °C for 5 s and 60 °C for 30 s. Throughout all qPCR assays, the elongation factor 1 (*Hl*-EF1) gene was used as an internal control to normalize the qPCR results.

### 2.5. Primary Culture and Challenge of Coelomocytes

To understand the response pattern of *Hl*-RIPK5, *Hl*-RIPK7, and *Hl*-MLKL against environmental factors and pathogens, the primary cultured coelomocytes from sea cucumber (*H. leucospilota*) were prepared and utilized to evaluate the response, as previously described [27]. Briefly, the primary coelomocytes were cultured at 28 °C in Leibovitz’s L-15 culture medium (Invitrogen, Carlsbad, CA, USA). After 18 h of incubation, the cultured coelomocytes were challenged with LPS (10 µg/mL, Sigma-Aldrich, St. Louis, MO, USA), poly(I:C) (10 µg/mL, Sigma-Aldrich, St. Louis, MO, USA) or *Vibrio harveyi* (10^7^ CFU/mL), and the cells were harvested at 24 h post-administration.

### 2.6. Statistical Analysis

All results of qPCR assays were presented as the mean ± standard error (SEM) of three biological replicates. The obtained data of mRNA expression level were assessed using one-way ANOVA followed by Tukey’s test with GraphPad Prism 9.0 (GraphPad Software, San Diego, CA, USA), and bars with different superscripts indicate significant differences (*p* < 0.05), or extreme differences (*p* < 0.01).

## 3. Results

### 3.1. Screening of the Genes of the RIPK Family and MLKL

**Figure 1:**
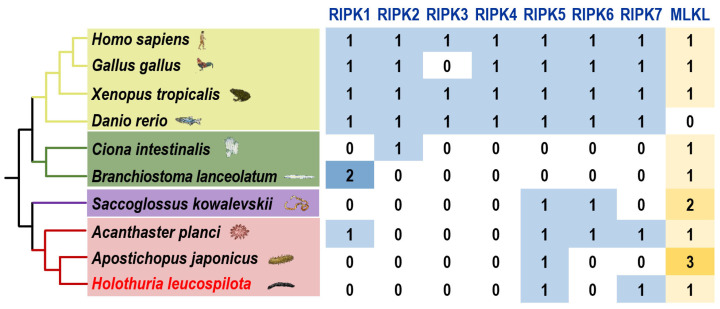
Numbers of the genes of the RIPK gene family and the MLKL gene among different species. In the evolutionary tree of 10 representative Deuterostomia species, the yellow branch represents Vertebrata, the green branch represents Urochordata and Cephalochordata, the purple branch represents Hemichordata and the red branch represents Echinodermata.

The result of genome-wide analysis reveals the presence of two members of the RIPK family (RIPK5 and RIPK7) and one MLKL gene in sea cucumber *H. leucospilota* (Figure 1). Additionally, cross-genome analysis showed that variation in the number and types of RIPK family genes among the analyzed ten Deuterostomia species (Figure 1), while MLKL was found to be highly conserved across these species (Figure 1).

The RIPK family is conserved in vertebrates (Figure 1), with all the members of the RIPK family (RIPK1–7) confirmed in vertebrate genomes, except for RIPK3, which is absent in the chicken genome. Hower, the member and numbers of the RIPK family are not conserved in Ambulacraria, with one to four members confirmed in their genomes. Specifically, only one member of the RIPK family was present in *B. lanceolatum* (*RIPK1*), *C. intestinalis* (*RIPK2*), and *A. japonicas* (*RIPK5*). Two RIPKs were identified in *S. kowalevskii* (*RIPK5*, *RIPK6*) and sea cucumber *H. leucospilota* (*RIPK5*, *RIPK7*). Additionally, four members were confirmed in *A. planci* (*RIPK1*, *RIPK5*, *RIPK6*, and *RIPK7*).

Unlike RIPKs, MLKL was confirmed in all analyzed species, with the exception of zebrafish *D. rerio*, where it was not detected. Furthermore, apart from *S. kowalevskii* and *A. japonicas*, which have two and three MLKL genes, respectively, all other species have only one MLKL gene (Figure 1).

### 3.2. Molecular Cloning and Sequence Analysis of Hl-RIPK5, Hl-RIPK7 and Hl-MLKL

Using RT-PCR, the *Hl*-RIPK5, *Hl*-RIPK7, and *Hl*-MLKL genes were successfully cloned and characterized from the sea cucumber *H. leucospilota*, and have been deposited in GenBank with the accession numbers PQ189392, PQ189393 and PQ189394, respectively. As shown in Figure S1, the ORFs of *Hl*-RIPK5, *Hl*-RIPK7 and *Hl*-MLKL ORF cDNAs are 2841 bp, 7875 bp and 1341 bp in length and are predicted to encode proteins of 946 amino acids (aa), 2625 aa and 426 aa, respectively.

The analyzed results from the InterPro grogram v5.27-66.0, SMART v9.0, and CD-search v3.21 indicate that the functional domain composition varies among RIPK5, RIPK7 and MLKL. As shown in Figure S2, a conserved kinase domain (residues 606–909) was predicted in the *Hl*-RIPK5 amino acid sequence; in the *Hl*-RIPK7 sequence, multiple domains were identified: an ANK domain (residues 621–706), LRR domains (residues 1082–1140 and 1177–1236), ROC domain (residues 1406–1593), COR domain (residues 1609–1825), another kinase domain (residues 1965–2217), and a WD40 domain (residues 2300–2423); additionally, a C-terminal pseudokinase domain (residues 174–431) was predicted in the *Hl*-MLKL amino acid sequence.

### 3.3. The Phylogenetic Tree and Functional Domain

The phylogenetic trees of the sea cucumber (*H. leucospilota*) RIPK family and MLKL were constructed separately with other nine representative species of Deuterostomia using MEGA X program. Details of the selected species and their protein are summarized in Table S2.

**Figure 2:**
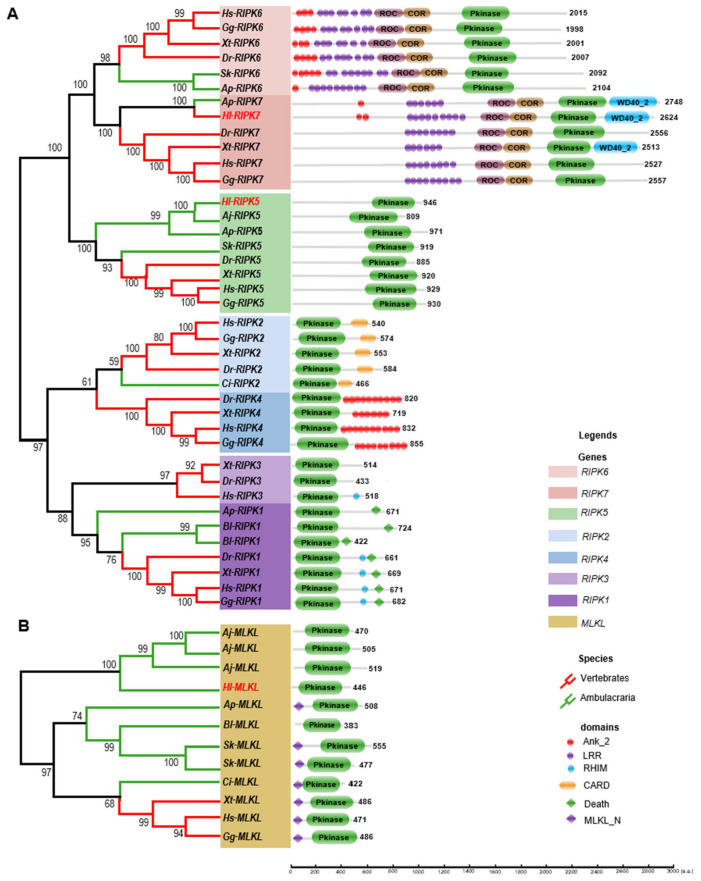
The phylogenetic tree and functional domain analysis of the RIPK family (**A**) and MLKL (**B**).

As illustrated in Figure 2A, the phylogenetic tree of the RIPK family is divided into two main branches, with RIPK5–7 proteins clustered in one branch and RIPK1–4 proteins clustered in the other. In the RIPK1–4 branch, RIPK1 and RIPK3 clustered together in one clade, while RIPK2 and RIPK4 formed a separate clade. Similarly, in the RIPK5–7 branch, RIPK6 and RIPK7 clustered in one clade, while RIPK5 formed its own distinct clade. The newly identified *Hl*-RIPK5 and *Hl*-RIPK7 in this study showed high homology to RIPK5 and RIPK7 from the sea cucumber *A. japonicas* and the starfish *A. planci,* respectively, and positioned outside the vertebrate RIPK5 and RIPK7 clusters. Furthermore, the distribution of RIPKs is uneven across the ten analyzed Deuterostomia species. RIPK5–7 are present in both vertebrates and Ambulacraria, while RIPK1–4 are primarily found in vertebrates, with exceptions in Ambulacraria, including *C. intestinalis* (Ci-RIPK2), *A. planci* (Ap-RIPK1), and *B. lanceolatum* (Bl-RIPK1).

To further elucidate the evolutionary relationships of the RIPK family, a functional domain analysis of RIPKs was performed across the ten Deuterostomia species, and the results were display in Figure 2A. A core conserved kinase domain was confirmed in all RIPKs across the ten representative species. In vertebrates, RIPK1 has a conserved kinase domain, a RHIM domain and a Death domain, while the RHIM domain was absence in Ambulacraria RIPK1, *Ap*-RIPK1 and *Bl*-RIPK1. The RIPK2 gene was found exclusively in Chordata, and it consistently features both a kinase domain and a CARD domain. RIPK3 was found only in human, frog and zebrafish. It has a conserved kinase domain, with the RHIM domain present only in human RIPK3. RIPK4 was only found in vertebrates with a conserved kinase domain, and C-terminal located several repeated Ank_2 domain was found.

RIPK5–7 genes were found in Deuterostomia, but are absent in Urochordata and Cephalochordata. All RIPK5 genes only have a conserved kinase domain without any extra functional domain. RIPK6 and RIPK7 genes are relatively large, encoding proteins with lengths of more than 2000 amino acids, with the exceptions of chicken (*Gg*-RIPK6, 1998 aa). Both RIPK6 and RIPK7 contain a kinase domain, a COR domain, a ROC domain, and one or more repeated LRR domains located at the N-terminal. In RIPK6, one or more ANK_2 domains are positioned before the LRR domains. In contrast, RIPK7 generally lacks ANK_2 domains, with the exceptions of *Ap*-RIPK7, which contains one ANK_2 domain, and *Hl*-RIPK7, which contains two ANK_2 domains. Additionally, some RIPK7 have a WD40_2 domain in C-terminal, including starfish *A. planci*, sea cucumber *H. leucospilota*, and frog *X. tropicalis*.

As the phylogenetic tree illustrated in Figure 2B, only one MLKL from sea cucumber (*H. leucospilota*) was confirmed and it clustered with two MLKLs from another sea cucumber (*A. japonicus*). All 12 MLKLs across ten species have a conserved kinase domain and a N-terminal MLKL-N domain, with the exception of sea cucumber (*H. leucospilota* and *A. japonicus*) and amphioxus (*B. lanceolatum*). According to the tree, four MLKLs form two sea cucumber species clustered into one single clade, the MLKLs form vertebrates clustered into one clade, and MLKLs form residual deuterostome clustered into one single clade. Surprisingly, the MLKL from sea squirt (*C. intestinalis*) clustered with those of vertebrates.

### 3.4. The Motif Patterns Analysis of RIPK5, RIPK7, and MLKL

**Figure 3:**
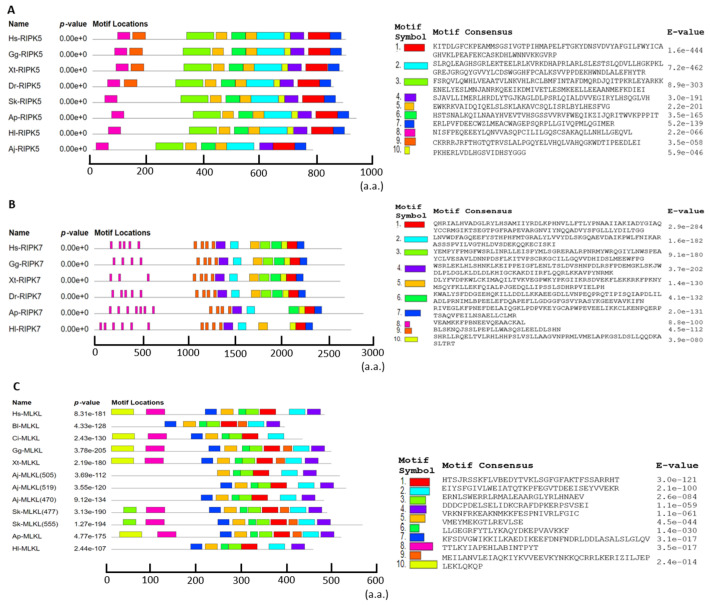
The protein motif patterns of RIPK5 (**A**), RIPK7 (**B**), and MLKL (**C**).

The results of the motif analysis were illustrated in Figure 3. The expected motif lengths were set between 6 and 100 amino acids, and the top ten motifs of RIPK5, RIPK7, and MLKL were selected and illustrated in Figure 3.

As illustrated in Figure 3, motif prediction and analysis revealed distinct structures and arrangements of motifs among RIPK5, RIPK7, and MLKL (Figure 3). In deuterostomes, RIPK5 generally has similar motif structures and arrangements, with the main difference being at the N-terminal, where vertebrates have a single additional motif 9 compared to invertebrates (Figure 3A). RIPK7 from both vertebrates and invertebrates showed a similar pattern, but RIPK7 from starfish and sea cucumber lacked two motifs at the C-terminal (Figure 3B). In MLKLs, motifs 1–6 were present as single copies, except for motif 3, which appeared as a double copy in *Sk*-MLKL (477 aa) and *Sk*-MLKL (555 aa). Motif 8 was present as a single copy in most MLKLs but was absent in the *Hl*-MLKL, *Aj*-MLKL, and *Bl*-MLKL proteins. Similarly, motif 10 was present as a single copy in most MLKLs but was absent in the *Hl*-MLKL, *Aj*-MLKL, *Bl*-MLKL, and *Sk*-MLKL proteins (Figure 3C).

### 3.5. mRNA Expression Patterns of Hl-RIPK5, Hl-RIPK7, and Hl-MLKL in Adult Tissues and Different Developments

**Figure 4:**
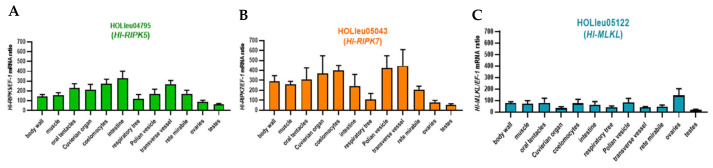
The mRNA expression profiles of *Hl*-RIPK5 (**A**), *Hl*-RIPK7 (**B**), and *Hl*-MLKL (**C**) in various adult tissues of the sea cucumber *H. leucospilota*. Bars represent the mean ± SEM (*n* = 3).

The spatial and temporal expression patterns of *Hl-RIPK5*, *Hl-RIPK7*, and *Hl-MLKL* were analyzed using qPCR across various adult tissues, as well as developing embryos and larvae of the sea cucumber (*H. leucospilota*) (Figure 4).

**Figure 5:**
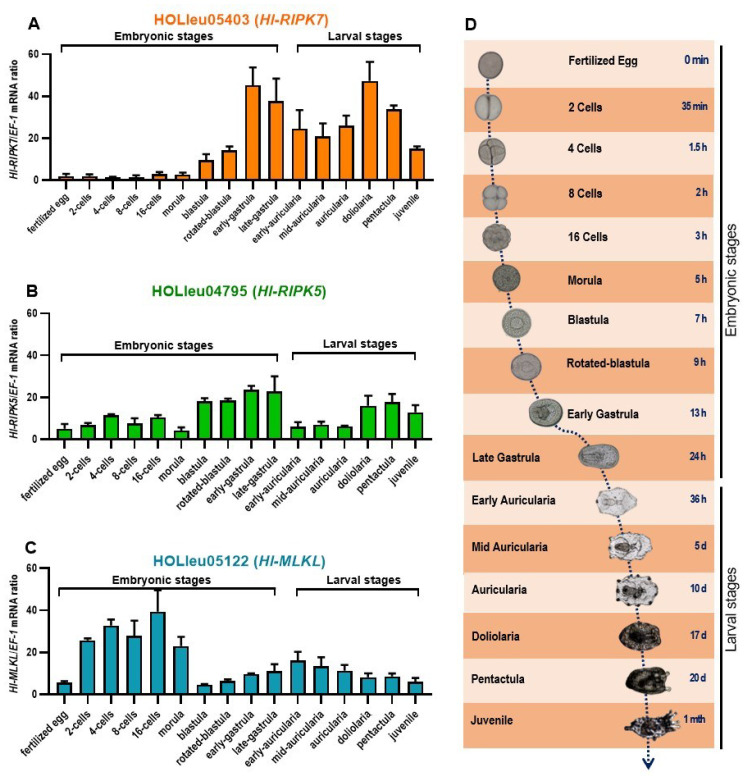
The mRNA expression profiles of *Hl-RIPK5* (**A**), *Hl-RIPK7* (**B**), and *MLKL* (**C**) in developing embryos and larvae of the sea cucumber *H. leucospilota*. (**D**) Typical embryonic and larval development of *H. leucospilota*. Numbers indicate time lapsed post-fertilization. Bars represent the mean ± SEM (*n* = 3).

The tissues tested included the body wall, muscle, oral tentacles, Cuvierian organ, respiratory tree, Polian vesicle, coelomocytes, intestine, transverse vessel, rete mirabile, ovaries, and testes. As illustrated in Figure 4, *Hl*-RIPK5 and *Hl*-RIPK7 share similar expression profiles, while *Hl*-MLKL exhibits a distinct pattern. As shown in Figure 5A,B, the *Hl*-RIPK5 and *Hl*-RIPK7 genes were detected in all tested tissues, with relatively low expression in the respiratory tree, ovaries, and testes. *Hl*-RIPK7 exhibited relatively high expression levels in the transverse vessel, Polian vesicle, and coelomocytes, while *Hl*-RIPK5 showed higher expression in the intestine, coelomocytes, and transverse vessel. Compared to RIPKs, the *Hl*-MLKL gene maintains a low transcriptional level in all the tissues examined, except for a high expression level in the ovary (Figure 4C).

RIPKs and MLKL exhibit distinct transcriptional patterns at embryonic stages and larval stages (Figure 5). Throughout embryonic development, the transcriptional expression level of *Hl*-RIPK7 varies across early, middle, and late stages (Figure 5A). At the early embryos, from the fertilized egg phase to the morula, the mRNA expression level of *Hl*-RIPK7 was low, with almost no obvious transcriptional activity (Figure 5A). In the late embryonic stages, the *Hl*-RIPK7 expression levels increased significantly, particularly during the early-gastrula and subsequent developmental stages, with peaks observed in the early-gastrula and the doliolaria stages (Figure 5A). During the larval stages, *Hl*-RIPK7 maintains relatively high transcriptional levels, peaking at the doliolaria stage (Figure 5A). *Hl*-RIPK5 transcriptional levels were detected throughout all embryonic and larval stages (Figure 5B). During the embryonic stages, its levels gradually increased, reaching a peak at the late-gastrula stage. During the larval stages, higher expression levels were observed at the doliolaria, pentactula, and juvenile stages.

As shown in Figure 5C, the mRNA expression pattern of *Hl*-MLKL differs from that of *Hl*-RIPK5 and *Hl*-RIPK7. The transcriptional activity of *Hl*-MLKL was observed across embryonic and larval stages of sea cucumber *H. leucospilota*. The *Hl*-MLKL mRNA expression was notably higher during the early embryonic stages, particularly from the 2-cell stage to the morula stage. From the late embryonic stage to the larval stages, *Hl*-MLKL exhibited an expression pattern that initially increased and then decreased, peaking at the early-gastrula stage. This pattern began at the blastula stage and persisted through to the juvenile stage.

### 3.6. Primary Coelomocytes Transcript Response of Hl-RIPKs and Hl-MLKL during Environmental and Pathogenic Challenges

**Figure 6:**
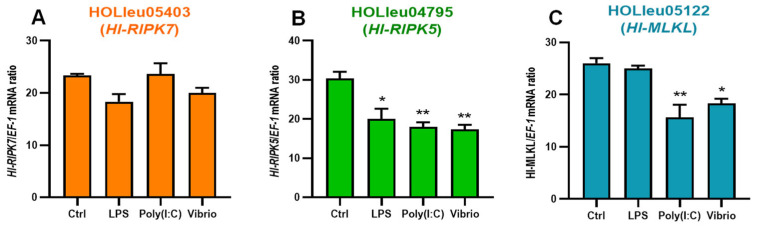
The transcriptional expression patterns of three *H. leucospilota* genes were examined following pathogenic and environmental challenges, including *V. harveyi* (10^7^ cells/mL), LPS (10 mg/mL), and poly(I:C) (10 mg/mL). The genes analyzed were HOLleu05403 (*Hl*-RIPK5) (**A**), HOLleu04795 (*Hl*-RIPK7) (**B**), and HOLleu05122 (*Hl*-MLKL) (**C**). *: *p* < 0.05 relative to Ctrl; **: *p* < 0.01 relative to Ctrl. Bars represent the mean ± SEM (*n* = 3).

Transcriptional activities were detected in cultured primary coelomocytes of sea cucumber following pathogenic challenges and environmental stresses. In this assay, the transcriptional responses of *Hl*-RIPK5, *Hl*-RIPK7 and *Hl*-MLKL exhibited distinct patterns in response to challenges with LPS, poly(I:C), or *V. harveyi* (Figure 6). The mRNA expression levels of *Hl*-RIPK7 were not significant variations under the stresses with LPS, poly(I:C), or *V. harveyi* (Figure 6A). The transcript response of *Hl*-RIPK5 following the challenge with LPS exhibited a significant decreased (*p* < 0.05), while its response to poly(I:C) and *V. harveyi* showed extremely decreased (*p* < 0.01) (Figure 6B). As shown in Figure 6C, there was no significant difference between the control and LPS challenge groups, while the *V. harveyi* and poly(I:C) challenge groups exhibited significant and extremely significant decreases, respectively.

## 4. Discussion

As important sensors for intracellular and extracellular stress [29], the RIPK family plays a crucial role in the innate immune system, serving as key modulators of inflammation, initial the necroptosis [3]. All members of the RIPK family (RIPK1–7) have been clearly found in vertebrates [29]. However, only a few are present in invertebrates, as RIPKs are fast-evolving and challenging to identify.

In the present study, genome-wide analysis was performed, and two members of the RIPK family (*Hl*-RIPK5 and *Hl*-RIPK7) and the *Hl*-MLKL gene were identified from sea cucumber (*H. leucospilota*). These were then compared with those of other deuterostomes (Figure 1).

Similar to their counterparts in other deuterostomes [29], *Hl*-RIPK5 contains a conserved C-terminal kinase domain and a large unknown function N-terminal sequence (Figure S1A). In contrast, *Hl*-RIPK7 was characterized by a C-terminal kinase domain and WD40 domain, along with N-terminal leucine-rich repeats, ankyrin repeats, ROC, and COR domains (Figure S1B). RIPK1–RIPK4 were present in vertebrates and chordates [25], but no corresponding members were identified from the sea cucumber (*H. leucospilota*). RIPK5–RIPK7 appears in both invertebrates and vertebrates, while RIPK1–RIPK4 was primarily found in vertebrates (Figure 2A). This suggested that RIPK5–RIPK7 may be more evolutionarily conserved genes within the RIPK family. It is worth mentioning that the RIPK1 found in amphioxus (*B. lanceolatum*) and starfish (*A. planci*) lacks the RHIM domain (Figure 2A), which is normally responsible for mediating necroptosis in vertebrates [30]. Deletion of this RHIM domain in the amphioxus and starfish indicates that different signaling pathways relative to necroptosis may be present in the immune response of invertebrates.

MLKL is ubiquitous and highly conserved in vertebrates and other deuterostomes [25], with some gene duplication observed in hemichordate and echinoderms (Figure 1). In this study, only one single MLKL gene (*Hl*-MLKL) was confirmed from sea cucumber (*H. leucospilota*), which contained a conserved pseudokinase domain (Figure S1C), but lacked the N-terminal MLKL_N domain. Similar structures were presented in the MLKL protein sequences of sea cucumber (*A. japonicus*) and Amphioxus (*B. lanceolatum*).

The MLKL_N-terminal domain forms a four-helical up-and-down bundle that is sufficient to induce liposome leakage and is crucial for necroptosis [31]. Additionally, homologs of the upstream kinases, RIPK1 and RIPK3 which form a complex called necrosome together with MLKL to induce necrosis in vertebrates [11,32]. Due to the absence of RIPK1 and RIPK3 in sea cucumber (*H. leucospilota*), and the lack of the MLKL_N domain in the *Hl*-MLKL protein, it is uncertain whether the *Hl*-MLKL is involved in mediating necroptosis or induces necroptosis through an alternative pathway. In summary, there are clear orthologous relationships between the RIPK family and MLKL in sea cucumbers and mammalian lineages.

RIPKs, particularly RIPK1, RIPK2, and RIPK3, have emerged as key regulators of inflammatory signaling and cell death pathways [3]. And activated MLKL is essential for the final steps of necroptosis, making it a crucial player in this cell death pathway [5]. Previous studies have indicated that the RIPK1–RIPK3–MLKL complex is crucial for inducing necroptosis [4], while RIPK2 plays a significant role in the activation of apoptosis [3]. Moreover, the RHIM domain in RIPK1 or RIPK3 is essential for their docking with MLKL [33,34]. In this study, RIPK1 and RIPK3 were not identified in sea cucumber (*H.* leucospilota); instead, RIPK5 and RIPK7, which lack the RHIM domain, were detected. Therefore, it remains unclear whether the RIPK family in sea cucumbers (*H. leucospilota*) is involved in necroptosis or if another pathway mediates necroptosis.

Phylogenetic analysis showed that RIPK5–RIPK7 clustered into one main branch, while RIPK1–RIPK4 clustered into another main branch, which is similar to the results in lamprey [29]. However, in our studies, RIPK1 and RIPK3 clustered into one clade, while RIPK2 and RIPK4 clustered into another clade. In the studies in lamprey, RIPK2-4 clustered into one clade, while RIPK1 clustered into another clade [29]. RIPKs are known for their regulatory roles in the innate immune system, which is facilitated by their conserved kinase domain and other functional domains within the DD superfamily, including the Death domain and the CARD domain [35] (Figure 2A). The conserved functional MLKL_N domain was observed in most MLKLs except in sea cucumber and Amphioxus (Figure 2B).

The motif patterns of RIPK5, RIPK7, and MLKL were different (Figure 3). RIPK5 and RIPK7 from different species exhibit high similarity in their motif patterns, suggesting that RIPK5 and RIPK7 have similar abilities for forming higher domain structures among deuterostomes, respectively. However, the motif patterns of MLKLs exhibited more variation between invertebrates and vertebrates, suggesting that these different motifs may have been involved in forming more various functional domains in deuterostomes.

*Hl*-RIPK5 and *Hl*-RIPK7 have similar tissue expression patterns while having different embryonic and larval developmental expression patterns (Figure 4A,B). Both *Hl*-RIPK5 and *Hl*-RIPK7 are expressed highly in coelomocytes which are considered the primary effector cells with phagocytosis in the echinoderms immune system [36]. Therefore, it can be speculated that *Hl*-RIPK5 and *Hl*-RIPK7 may be immune defense-related genes in sea cucumber. In lamprey, both RIPK5 and RIPK7 are also mainly expressed in immune-related tissues, such as leukocytes [29]. And *Hl*-RIPK7 is considerably higher than *Hl*-RIPK5 in the adult tissues investigated, which is similar to the results in lamprey [29]. In mammals, RIPK7 is expressed in various tissues, with high expression in the brain, lung, kidney and peripheral immune cells such as B lymphocytes, monocytes and neutrophils [37]. The hypothesis that human RIPK7 plays an important role in the innate immune inflammatory pathways is further strengthened by findings that RIPK7 polymorphisms enhance the risk of developing PD and inflammatory bowel disease [38,39,40,41].

For the embryonic developmental expression study, we found that the mRNA expression levels of *Hl*-RIPK5 and *Hl*-RIPK7 expressed at high levels at blastula, gastrula and larval stages (Figure 5A,B). The nerves of the sea cucumber may have initially formed during the larval stages, as reported in *A. japonicus*, with the development of five radially symmetrical nerve structures at the base of the oral tentacles [42]. In addition, the intestine of sea cucumbers gradually matures at the larval stages, and they need to accumulate nutrition for the transformation of planktonic to benthic lifestyles [28,43]. Hence, the roles of *Hl*-RIPK5 and *Hl*-RIPK7 at the embryonic and larval stages are speculated to be related to neurogenesis and intestinal development. In mammals, RIPK7 was thought to play an important role in controlling proliferation, migration, and differentiation of neural cells as well as in morphogenesis of extra-neural tissues. RIPK7 mRNA is detectable at E8.5 in non-neural and at E10.5 in neural tissues in mouse [44]. In non-neural tissues, mouse RIPK7 was highly expressed in limb interdigital zones, developing kidney glomeruli, and spermatogenetic cells [44].

Mouse MLKL are widely expressed in various tissues including spleen, kidney, liver, lung, heart, brainstem, frontal cortex and spinal cords, but absent form CNS tissues [45]. The data of human protein atlas project (https://www.proteinatlas.org/, accessed on 1 September 2022) reveal that MLKL mRNA levels are the highest in bone marrow and immune system. IFN signaling induces MLKL mRNA and protein expression in breast carcinoma and Hela carcinoma cells [46]. Conditions of inflammation and tissue injury can also upregulate the expression of MLKL [47]. In the present study, we identified one MLKL gene in the genome of sea cucumber (*H. leucospilota*), and the transcripts of *Hl*-MLKL were detected in all the tested tissues, with the highest expression level in the ovary. Hence, we speculated the function of *Hl*-MLKL may be related to ovarian development. MLKL is reported to be involved in differentiation during embryonic development in vertebrates. MLKL is thought to be dispensable for normal mouse development as well as immune cell development, though the MLKL-deficient mice are viable, healthy, fertile and do not show any abnormities in development [48]. Our present study showed that the *Hl*-MLKL gene expressed highly from the 2-cell stage to the morula stage, then remained a low level for the subsequent stages (Figure 5C). From the 2-cell stage to the morula stage, many biological processes including cell differentiation, cell apoptosis, cell movement, actin cytoskeleton, autophagy and endocytosis are involved [28]. In mammals, MLKL interacts with RIPK3 to execute necroptosis; however, we did not find RIPK3 in sea cucumber genome. Given that apoptosis is a complicated mechanism that can be mediated by multiple pathways, it is possible that *Hl*-MLKL are speculated to regulate cell death in the embryonic stages through a pathway different from its mammalian counterparts.

Coelomocytes are the effector cells of the echinoderm immune system capable of responding to injury or infection and playing a role in the clearance of foreign substances and bacteria [36]. Primary coelomocytes are considered effective research tools for investigating the responses of sea cucumbers to pathogens and environmental stresses. In our previous studies [18,49], we examined the transcriptional patterns of genes related to the intrinsic apoptotic pathway and the responses of caspases to pathogens, environmental factors, LPS, and various compounds.

The mRNA expression of *HI*-RIPK5, *Hl*-RIPK7, and *Hl*-MLKL was observed in the coelomocytes of sea cucumber (*H. leucospilota*) in this study (Figure 4C). These results indicated that *Hl*-RIPK5, *Hl*-RIPK7, and *Hl*-MLKL may be involved in the immune response in sea cucumber coelomocytes. In cultured primary coelomocytes, *Hl*-RIPK5 expression was significantly depressed with the challenges of LPS, poly(I:C), or pathogen *V. harveyi*. In contrast, *Hl*-RIPK7 expression levels were unchanged following the same challenges (Figure 6A,B). Similar to the *Hl*-RIPK5, the *Hl*-MLKL mRNA levels were significantly decreased with poly(I:C) or *V. harveyi*, but did not change with LPS (Figure 6C). In summary, RIPK5 and MLKL, rather than RIPK7, may be the key regulators of the sea cucumber immune system. They could be crucial modulators in determining cell fate under stress or infection, guiding the cells towards apoptosis, necrosis, or immune activation depending on the environment and stimuli.

## 5. Conclusions

In this study, RIPK5, RIPK7, and MLKL were identified in the sea cucumber (*H. leucospilota*). *Hl*-RIPK5 and *Hl*-RIPK7 were highly expressed in the coelomocytes of adult sea cucumbers, while *Hl*-MLKL showed its highest expression in the ovaries. *Hl*-RIPK7 mRNAs were predominantly expressed from the morula stage to the juvenile stage, with *Hl*-RIPK5 primarily expressed during the late embryonic and late larval stages. *Hl*-MLKL was mainly expressed from the 2-cell stage to the morula stage. In primary coelomocytes, the expression of RIPK5 and MLKL was significantly downregulated under pathogenic or environmental stress, while RIPK7 expression remained unchanged. However, the differences in developmental stages across species could have influenced these comparisons, as gene functions may vary significantly depending on the stage of development. For example, *Hl*-RIPK5 and *Hl*-RIPK7 showed high expression during blastula, gastrula, and larval stages, which may be linked to neurogenesis and intestinal maturation in sea cucumbers. In contrast, the roles of RIPK7 in mammals have been characterized at different embryonic stages and in different tissues. In future studies, it is essential to ensure that comparisons are made between similar developmental stages to enhance the reliability of functional interpretations. In summary, our study is crucial for elucidating the role of the RIPK family and MLKL in sea cucumbers, particularly in understanding their response to pathogens. These findings lay a foundation for future research into molecular breeding and the innate immune system of sea cucumbers.

## Data Availability

Data will be made available on request.

## Supplementary Materials

The following supporting information can be downloaded at: https://www.mdpi.com/article/10.3390/genes15101297/s1, Table S1: Primers used for ORF cloning and qPCR. Table S2: Information of species and peptide sequences of the RIPK gene family and MLKL used for analysis. Figure S1: Numbers of RIPK genes of the RIPK gene family and the MLKL gene among different species. In the evolutionary tree of 10 representative Deuterostomia species, the yellow branch represents Vertebrata, the green branch represents Urochordata and Cephalochordata, the purple branch represents Hemichordata and the red branch represents Echinodermata.
