# Supplementary material for: Genomic and Transcriptional Analysis of the Necroptosis Pathway Elements RIPK and MLKL in Sea Cucumber, Holothuria leucospilota
Source: Genes (Basel). 2024 Oct 3;15(10):1297. doi: 10.3390/genes15101297 (PMC11507063; doi:10.3390/genes15101297)
Supplement: Supplementary file 1 [file genes-15-01297-s001.zip › Figure S1.PPT]

## Slide 1
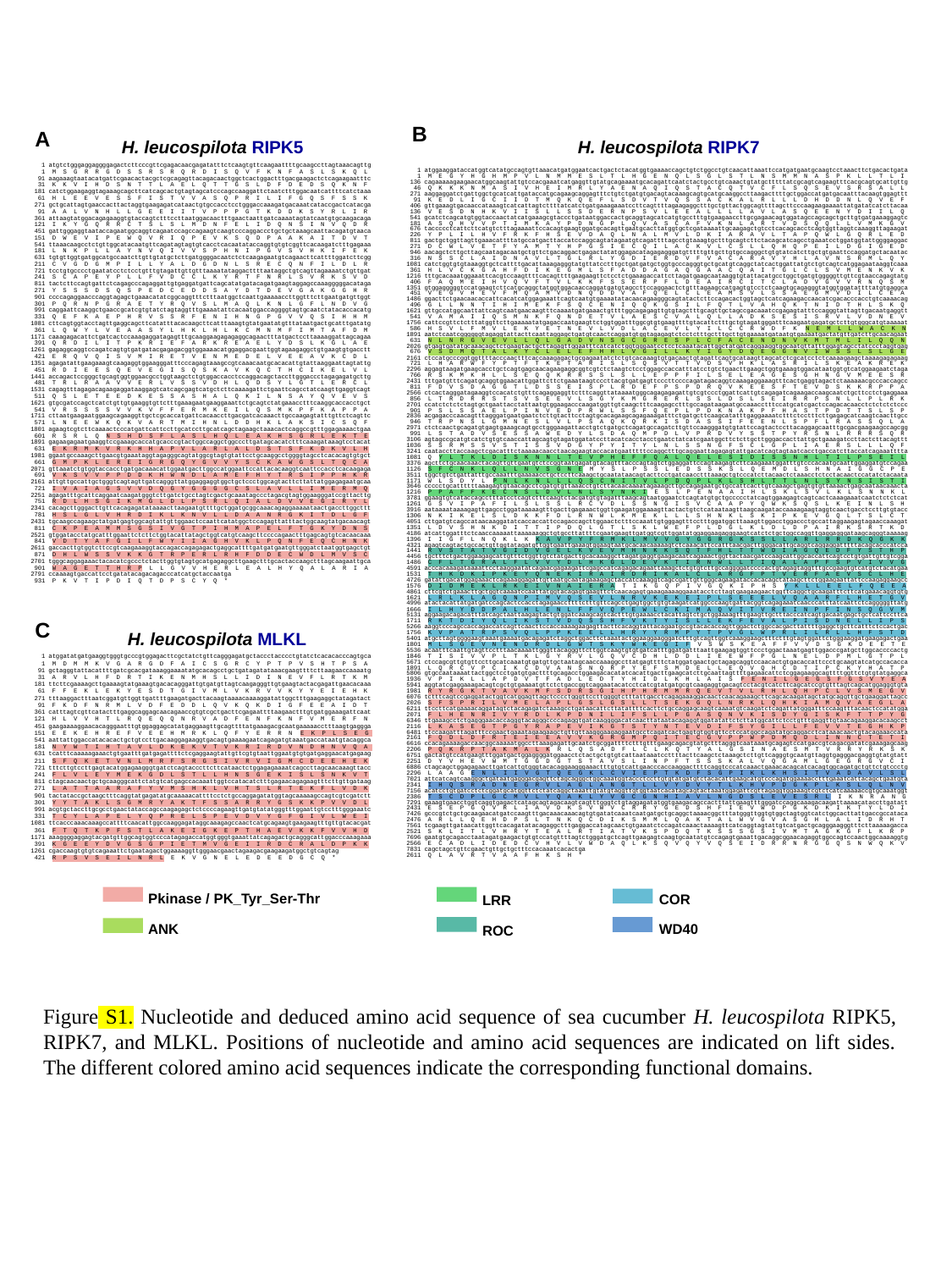

B
A
H. leucospilota RIPK5
H. leucospilota RIPK7
 1 atggaaggataccatggtcatatgccagtgttaaacatgatggaatcactgactctacatggtgaaaaccagctgtctggcctgtcaacattaaattccatgatgaatgcaagtcctaaacttctgacactgata
 1 M E G Y H G H M P V L N M M E S L T L H G E N Q L S G L S T L N S M M N A S P K L L T L I
 136 cagaaaaagaagaacatggcaagtattgtccacgaaatcatgaggttgtatgcagaaaatgcacagattcaatctactgcctgtcaaactgtatgctttttatcgcagtcagaagtttcacgcagtgcattgttg
 46 Q K K K N M A S I V H E I M R L Y A E N A Q I Q S T A C Q T V C F L S Q S E V S R S A L L
 271 aaggaggatctgattggctgcatcattgataccatgcagaagcaggagtttctgtctgatgtgacagtacaaagcagtgcatgcaaggccttaagacttttgctggaccatgatgacaatttacaagtggagttt
 91 K E D L I G C I I D T M Q K Q E F L S D V T V Q S S A C K A L R L L L D H D D N L Q V E F
 406 gttgaaagtgacaaccataaagtcatcattagtcttttatcatctgatgaaagaaatccttcagttttagaagaggctttgctgttactggcagttttagcttcccaagaagaaaattatgatatcatcttacaa
 136 V E S D N H K V I I S L L S S D E R N P S V L E E A L L L L A V L A S Q E E N Y D I I L Q
 541 gcatctcagcatgtggtaccaactatcatgaaagcgtaccctgataatggaccactgcaggtagcatcatgtgcctttgtgaagaaccttgcgagaacagtggatagccagcagctgcttgtgatgaaaggagtc
 181 A S Q H V V P T I M K A Y P D N G P L Q V A S C A F V K N L A R T V D S Q Q L L V M K G V
 676 taccccctcattcttcatgtctttagaaaattccacagtgaagtggatgcacagttgaatgcacttatggtgctcgataaaattgcaagagctgtcctcacagcacctcagtggttaggtcaaaggttagaagat
 226 Y P L I L H V F R K F H S E V D A Q L N A L M V L D K I A R A V L T A P Q W L G Q R L E D
 811 gactgctggttagttgaaacattttatgccatgacttaccatccaggcagtatagaatgtcagattttagcctgtaaagtgctttgcagtcttctacagcatcagcctgaaatcctggatggtattggggaggac
 271 D C W L V E T F Y A M T Y H P G S I E C Q I L A C K V L C S L L Q H Q P E I L D G I G E D
 946 aacagctcttgcttagcaatagacaatgctgttctgacaggactgagactatatggagacatagagagggatgtttttgttgcttgtgccagggctgtgtatcatcttgctgtgaattccaggatgctacaatac
 316 N S S C L A I D N A V L T G L R L Y G D I E R D V F V A C A R A V Y H L A V N S R M L Q Y
1081 catctggtgtgtaaaggtgctcattttgacattaaagagggtatgttatcctttgctgatgatgctggtgcccagggtgctgcatgtcaggctatcactggattatgtctgtcagtcatggagaataaggtcaaa
 361 H L V C K G A H F D I K E G M L S F A D D A G A Q G A A C Q A I T G L C L S V M E N K V K
1216 tttgcacaaatggaaattcacgtccaagttttcacagttttgaagaagttctcctctgaaagaccattcttagatgaagcaataaggtgtattacatgcctggctgatgtgggggttgttcgtaaccagagtatg
 406 F A Q M E I H V Q V F T V L K K F S S E R P F L D E A I R C I T C L A D V G V V R N Q S M
1351 gtggagggggtccatgaagtcttcatgcaggctatggtggacaaccaggatgatgtagccttccaggaactctgtttagaagccatgagtgtcctctcaagtgcagagggtatggtggatattttatgtgaggca
 451 V E G V H E V F M Q A M V D N Q D D V A F Q E L C L E A M S V L S S A E G M V D I L C E A
1486 ggacttctgaacaacaccattcacatcatggagaaattcagtcaatgtgaaaatatacaacagaagggcagtatactcttccagacactggtagctcatcagaagaccaacatcgacacccacctgtcaaaacag
 496 G L L N N T I H I M E K F S Q C E N I Q Q K G S I L F Q T L V A H Q K T N I D T H L S K Q
1621 gttgccatggcaattattcagtcaatgaacaagtttcaaaatgatgaaactgttttggcagagagttgtgtagctttgcagttgctagccgacaaatccgagagtatttccagggtattagttgacaatgaggtt
 541 V A M A I I Q S M N K F Q N D E T V L A E S C V A L Q L L A D K S E S I S R V L V D N E V
1756 cattccgttctctttatggttcttgaaaaatatgagaccaatgaagttctggtggatttggcgtgtgaagttttgtacattctttgctgtagatgggatttcaagaatgagatgctcttgtgggcatgtaaaaat
 586 H S V L F M V L E K Y E T N E V L V D L A C E V L Y I L C C R W D F K N E M L L W A C K N
1891 aatctcaatcggggggtagaggtattacttcaattaggagctgatgtcaactcaggatgtggtagagagtctcctctttgctttgcttgtgaaaatgataatgtgaaaatgactatgttgatcttgcaacagaat
 631 N L N R G V E V L L Q L G A D V N S G C G R E S P L C F A C E N D N V K M T M L I L Q Q N
2026 gtgagtgatatgcaaacagctctgaagtactgcttagagttggaatttcatcatctggtgggaatcctcctcaaatacattggctatgatcaggaaggtggcaatgttatttggagtagcctatccctaggtgag
 676 V S D M Q T A L K Y C L E L E F H H L V G I L L K Y I G Y D Q E G G N V I W S S L S L G E
2161 ctccatgcccggtggttttacccaactttcaccaaagagactgcgagaatatctctgtcacaaagtgtgacaactgtagattcagtgcataagttagcatctcgcatcctctcaaaagaagctaaaagagagaag
 721 L H A R W F Y P T F T K E T A R I S L S Q S V T T V D S V H K L A S R I L S K E A K R E K
2296 aggagtaagatgaagcacctgctcagtgagcaacagaagaggcggtcgtctctaagtctcctggagccaccatttatcctgtctgaacttgaagctggtgaaagtggacataatggtgtcatggaagaatctaga
 766 R S K M K H L L S E Q Q K R R S S L S L L E P P F I L S E L E A G E S G H N G V M E E S R
2431 tttgatgtttcagatgcaggtggaacattggattcttctgaaataagtcccttacgtgatgagttcccttccccagatagacaggtcaaagaggaaagtttcactgaggtagactctaaaaaacgcccaccagcc
 811 F D V S D A G G T L D S S E I S P L R D E F P S P D R Q V K E E S F T E V D S K K R P P A
2566 ctcactagggatagaaggtccacatctgtttcagaggaggttctttcaggttataaaatgggcagagagagattgtcgtccctggattcattgtcagagatcagaagaccaagcaatctgcttcctctgaggaaa
 856 L T R D R R S T S V S E E V L S G Y K M G R E R L S S L D S L S E I R R P S N L L P L R K
2701 ccatctctctctagtgctgaattacctattaatgtggaagacccaagatggttgtcaagctttcaagagcctttgccagataagaatgccaaacctttccatgcatcgactccagacacaacctctctctctccc
 901 P S L S S A E L P I N V E D P R W L S S F Q E P L P D K N A K P F H A S T P D T T S L S P
2836 acgagacccaacagtttagggatgaatgaatctcttgtacttcctagtgcacagaagcagagaaagatttctgatgcttcaagcatatttgaggaaaatctttctcctttcttgagagcatcaagtcaacttgcc
 946 T R P N S L G M N E S L V L P S A Q K Q R K I S D A S S I F E E N L S P F L R A S S Q L A
2971 ctctcaactgcagatgtgagtgaaagcagtgcctgggaagattacctgtctgatgctcagatgccagatcttgttccaagggatgtgtattccagtactccttacaggagcaatttgcgacgaagaagccagcgg
 991 L S T A D V S E S S A W E D Y L S D A Q M P D L V P R D V Y S S T P Y R S N L R R R S Q R
3106 agtagccgcatgtcatctgtgtcaaccattagcagtgtagatggatatccttacatcacctacctgaatctatcatcgaatggcttctcttgcttgggaccacttattgctgaaagatccttactcttacagttt
1036 S S R M S S V S T I S S V D G Y P Y I T Y L N L S S N G F S C L G P L I A E R S L L L Q F
3241 caataccttaccaagctcgacatttctaaaaacaacctaacagaagtaccacatgaatttttccaggctttgcaggaattagagagtattgacatcagtagtaatcacctgaccatcttaccatcagaaatttta
1081 Q Y L T K L D I S K N N L T E V P H E F F Q A L Q E L E S I D I S S N H L T I L P S E I L
3376 agcttctgcaacaaactacagttgttgaatgtctccggtaatgagatgtacagtttacccagtagtctggaggattccagtaagagtcttcaagaaatggatttgtcccacaatgcaattggaggatgtccagaa
1126 S F C N K L Q L L N V S G N E M Y S L P S S L E D S S K S L Q E M D L S H N A I G G C P E
3511 tggctgtctgattatttgccaaatttgaaaaacctgctccttcaaagctgcaatataacagtacttcctgatcaacctttaaagctgtcccatcttacaactctaaacctctcctacaactccatatctacaata
1171 W L S D Y L P N L K N L L L Q S C N I T V L P D Q P L K L S H L T T L N L S Y N S I S T I
3646 ccccctgcattttttaaagagtgtaacagcctcgatgtgttaaacctgtcttacaacaaaatagaaagcttgccagagaatgctgccattcacttgtcaaagctgagtgtgttaaaactgagcaataacaaacta
1216 P P A F F K E C N S L D V L N L S Y N K I E S L P E N A A I H L S K L S V L K L S N N K L
3781 ggaagtgtcataccagcctttatcctcagtctttcaagtctacgatgtgtagatttaagcagtaatggaatctcagtatgtgctgccccctatcagtggaagagtcagtcactcaaagaaatcaatctctctcat
1261 G S V I P A F I L S L S S L R C V D L S S N G I S V C A A P Y Q W K S Q S L K E I N L S H
3916 aataaaataaaagagttgagcctggataaaaagtttgacttgagaaactggttgaagatggaaaagttactactgtctcataataagttaagcaagataccaaaagaagtaggtcaactgacctccttgtgtacc
1306 N K I K E L S L D K K F D L R N W L K M E K L L L S H N K L S K I P K E V G Q L T S L C T
4051 cttgatgtcagccataacaaggatatcaccaccattccagaccagcttggaactctttccaaattgtgggagtttcctttggatggcttaaagttggacctggaccctgccattaggaagagtagaaccaaagat
1351 L D V S H N K D I T T I P D Q L G T L S K L W E F P L D G L K L D L D P A I R K S R T K D
4186 atcattggatttctcaaccaaaaattaaaaaaggctgtgccttattttcgaatgaagttgatggtcgttggatatggaggaagagggaagtcatctctgctggccaggttgaggagggataagcagggtaaaaag
1396 I I G F L N Q K L K K A V P Y F R M K L M V V G Y G G R G K S S L L A R L R R D K Q G K K
4321 agagtcagtactgccactgttggtatagatgttggtgaattgaaagtggaagtaatgcacaacaaaaagtctcaaacattccatttaacgacttgggacattgcaggtcaggaggatttttacagcacccatcca
1441 R V S T A T V G I D V G E L K V E V M H N K K S Q T F H L T T W D I A G Q E D F Y S T H P
4456 tgctttctgactggaagagcattgtttctggttgtctatgacttgcacaaaggcttagatgaggtgaagacaatcagaaactggttactaacgatccaagcattggcaccattcagtcctgtgattgttgtcgga
1486 C F L T G R A L F L V V Y D L H K G L D E V K T I R N W L L T I Q A L A P F S P V I V V G
4591 acccacaaagataaaattcctaaggaatatcagaacgagaagattcgagccatcagagacagaattaaagctctgtgtcttgccaggggatccccactgtagagtaggtttgccgaagtgtcatgtctacatgaa
1531 T H K D K I P K E Y Q N E K I R A I R D R I K A L C L A R G F P T V E R F A E V S C L H E
4726 gatattgacatggagaaactcagaaaggagattgttaatgcaatagaaagagctaccatcaaaggtcagccgattgttgggcagaagataccacacagctataagcttctggaagaattattccaagaggaagcc
1576 D I D M E K L R K E I V N A I E R A T I K G Q P I V G Q K I P H S Y K L L E E L F Q E E A
4861 cttcgtctgaaacttgctggtcaaaatccaattatggtacagagtgaagttctcaacagagtgaaagaaaaggaaatacctcttagtgaagaagaactggttcaggctgcaagatttcttcatgaaacaggtgtg
1621 L R L K L A G Q N P I M V Q S E V L N R V K E K E I P L S E E E L V Q A A R F L H E T G V
4996 atactacattatgatgatccagcactccacctagagaaccttttctttgttcagcctgagtggctgtgtaagatcatggcccaagtgattacggtcagagaaatcaacccatttatcaattctcagggggttatg
1666 I L H Y D D P A L H L E N L F F V Q P E W L C K I M A Q V I T V R E I N P F I N S Q G V M
5131 aggaagacagacatttatcagctaattaagagtactgtggatcaaagcagtcactttgtgaaaacctacattagtctgctggaaaagtttgaagttgctttacccatcagtgacaatgagctgctcattccttca
1711 R K T D I Y Q L I K S T V D Q S S H F V K T Y I S L L E K F E V A L P I S D N E L L I P S
5266 aaggtcccagccaccagaccatcagttcaacttccaccaaaagaagagttacttcacaggtattacagaatgccgtacacaccagttggactctggccacgacttattttgaggctgcttcatttctccactgac
1756 K V P A T R P S V Q L P P K E E L L H R Y Y R M P Y T P V G L W P R L I L R L L H F S T D
5401 atgcttagtggggaagtaaatgaaaatgacagagatctaggctggacttctaaatactggaaggaagggatctttgtcagttggtcaaaggaagctttctttgtagtggattctgggaaggatgaagagactgaa
1801 M L S G E V N E N D R D L G W T S K Y W K E G I F V S W S K E A F F V V D S G K D E E T E
5536 acaatttcaattgtagttcctttaacaaaattgggttacagggttcttggtcaagtgtgtgatcatttggatgatttaattgaagagtggttccctggactaaatgagttggacccgatgcttggcaccccactg
1846 T I S I V V P L T K L G Y R V L G Q V C D H L D D L I E E W F P G L N E L D P M L G T P L
5671 ctccagcgttgtgttccttgcatcaaatgtgatgttgctaatagcaaccaaaggccttatgagttttctatggatgaactgctagagcaggtccaacactgtgacaccattccctgcaagtatcatgccacacca
1891 L Q R C V P C I K C D V A N S N Q R P Y E F S M D E L L E Q V Q H C D T I P C K Y H A T P
5806 gtgccaataaaattactggctcctgatgtgacttttgcagacctggaagacacatatcacattgacttgaagcatcttgcaattagttttgagaacattctcggagaaggcagttttggttctgtgtatgaggca
1936 V P I K L L A P D V T F A D L E D T Y H I D L K H L A I S F E N I L G E G S F G S V Y E A
5941 aggtatcgaggaaagacagtcgctgtgaaaatgttctctgaccggtcaggaatacatcctcatcgtatgatgcgtcaagaggtgacagtcctacgtcatcttcagcatccgtgtttagtcagcatggagggtgta
1981 R Y R G K T V A V K M F S D R S G I H P H R M M R Q E V T V L R H L Q H P C L V S M E G V
6076 tctttcagtccgaggatactggtcatggagttagctcccctgggttccttggggtctttattgacctcagagaaaggacaactcaacagaaagcttcagcacaagatagctatgcaggttgctgaaggattagcc
2026 S F S P R I L V M E L A P L G S L G S L L T S E K G Q L N R K L Q H K I A M Q V A E G L A
6211 ttccttcatgaaaacaggatagtctacagagatctaaagcctgataacattcttatattttcacttctgccaggagcaagtcaaaatgtcaagatctcagattatgggatttccaagtttacaactccatatgga
2071 F L H E N R I V Y R D L K P D N I L I F S L L P G A S Q N V K I S D Y G I S K F T T P Y G
6346 ttgaaagcctctgagggaacaccagggtacagggccccagaggtgatcaaggggacatcaacttataatacagaggtggatatattctcttatggcattctcctgtttgaggttgtaacagaaggacacaagcct
2116 L K A S E G T P G Y R A P E V I K G T S T Y N T E V D I F S Y G I L L F E V V T E G H K P
6481 ttccaagatttagatttccgaactgaaatagaagaagctgttgttaagggaagaggaatgcctcagatcactgagtgtggtgttcctccatggccagatatgcaggacctcataaacaactgtacagaaaccata
2161 F Q D L D F R T E I E E A V V K G R G M P Q I T E C G V P P W P D M Q D L I N N C T E T I
6616 ccacagaaaagaccaacggcaaaaatggccttaaagagattgcaatctgcggattttctttgtttgaagcagacgtatgctttagggtcaataaatgcagagtccatgaccgtcagacgatatcgaaagagcaag
2206 P Q K R P T A K M A L K R L Q S A D F L C L K Q T Y A L G S I N A E S M T V R R Y R K S K
6751 gactatgtccatgaagtttggatgactggaggggatggaacctctactgctgtgtcattaataaatccttttacttcaagctctaaagctcttgttcagggtgcaatgttaggtgagggacgaggtgtatgtatt
2251 D Y V H E V W M T G G D G T S T A V S L I N P F T S S S K A L V Q G A M L G E G R G V C I
6886 ctagcagctggagagaacttgatcattgtgggtacacaggaagggaaactttgtgtcattgaacccaccaaggacttttcaggtcccatcaaactgaaacacagcatcacagtggcagatgctgttctgtccctg
2296 L A A G E N L I I V G T Q E G K L C V I E P T K D F S G P I K L K H S I T V A D A V L S L
7021 attcatcagtcaagggctgataatgagggacgagttctagcagggctggcaaatggtaccctccttgtgtatgatgtctacacattgaagcatgtcccagatggaaaacctttgaaatcattacagctgaatgta
2341 I H Q S R A D N E G R V L A G L A N G T L L V Y D V Y T L K H V P D G K P L K S L Q L N V
7156 acatcctgtgatcctctcggatgcatggttctctatcaggctaaattgtatgtaggttgtggtaatcacatagcagtactaaatggagattcgttagaggtggaaagtcgtctcatcaaaaaccgtgcaaatggt
2386 T S C D P L G C M V L Y Q A K L Y V G C G N H I A V L N G D S L E V E S R L I K N R A N G
7291 gaaagtgaacctggtcaggtgagactcatagcagtagacaagtcagtttgggtctgtaggagatatggtgaagacagccactttattgaagtttgggatccaggcaaagacaagattaaaacataccttgatatt
2431 E S E P G Q V R L I A V D K S V W V C R R Y G E D S H F I E V W D P G K D K I K T Y L D I
7426 gcccgtctgctgcaagaacatgatccaagtttgacaaacaaacagtgtgatatcaaatcaatgatgctgcaggctaaaacggctttatgggttggtgtggctagtggtcatctggcacttattgaccgccataca
2476 A R L L Q E H D P S L T N K Q C D I K S M M L Q A K T A L W V G V A S G H L A L I D R H T
7561 tcgaagttgataacattggttcacagatatacagaggctttgaggaccatagcaactgtgaaatctccagatcaaactaaaagttcatcaggtagtattgtcatgactgcagggaagggtttcttaaaaagacca
2521 S K L I T L V H R Y T E A L R T I A T V K S P D Q T K S S S G S I V M T A G K G F L K R P
7696 gaatgtgcagacctaatagatgaagactgtgtccatgttttagtctgggatgctcagttgaagagtcaagtgcaatatgtccagagtgaaattgacaggcggaacagaggtggccagtccaactggcaaaaggtg
2566 E C A D L I D E D C V H V L V W D A Q L K S Q V Q Y V Q S E I D R R N R G G Q S N W Q K V
7831 cagctagctgttcgaactgttgctgctttccacaaatcacactga
2611 Q L A V R T V A A F H K S H *
 1 atgtctgggaggaggggagactcttcccgttcgagacaacgagatatttctcaagtgttcaagaattttgcaagccttagtaaacagttg
 1 M S G R R G D S S R S R Q R D I S Q V F K N F A S L S K Q L
 91 aagaaagtaatacatgattcgaacactacgctcgcagagttacagacaactggctcactggactttgacgaagactctcagaagaatttc
 31 K K V I H D S N T T L A E L Q T T G S L D F D E D S Q K N F
 181 catctggaagaggtagaaagcagcttcatcagcactgtagtagcatcccagccaaggattctaatctttggacaatcattttcatctaaa
 61 H L E E V E S S F I S T V V A S Q P R I L I F G Q S F S S K
 271 gctgcattagtgaaccacttactaggtgaagagatcataactgtgccacctcctgggaccaaagatgacaaatcataccgactcatacga
 91 A A L V N H L L G E E I I T V P P P G T K D D K S Y R L I R
 361 attaagtatggacagagaaggtgtaccagtctttccttaatggacaactttgaactaattgatcaaaatagtatcaatgtgcaagacaga
 121 I K Y G Q R R C T S L S L M D N F E L I D Q N S I N V Q D R
 451 gattgggaggtaataccagaatggcaggtcagaatccagccagaagtcaagtcccaggaccctgctgctaaagcaattacagatgtaaca
 151 D W E V I P E W Q V R I Q P E V K S Q D P A A K A I T D V T
 541 ttaaacaagcctctgttggcatacaatgttcagatagtagtgtcacctcacaatataccaggtgtgtcggttcacaagatctttgagaaa
 181 L N K P L L A Y N V Q I V V S P H N I P G V S V H K I F E K
 631 tgtgttggtgatggcatgccaatcttgttgtatgctcttgatggggacaatctctcaagagaatgtcagaacttcattttggatcttcgg
 211 C V G D G M P I L L Y A L D G D N L S R E C Q N F I L D L R
 721 tcctgtgcccctgaatatcctctcctgtttgtagattgttgtttaaaatataggacttttaataggctgtcagttagaaaatctgttgat
 241 S C A P E Y P L L F V D C C L K Y R T F N R L S V R K S V D
 811 tactcttccagtgattctcagagcccagaggattgtgaggatgattcagcatatgatacagatgaagtaggagccaaagggggacataga
 271 Y S S S D S Q S P E D C E D D S A Y D T D E V G A K G G H R
 901 ccccagaggaacccaggtagagctgaaacatatcggcaggtttctttaatggctcaattgaaaaaccttggtttcttgaatgatgttggt
 301 P Q R N P G R A E T Y R Q V S L M A Q L K N L G F L N D V G
 991 caggaattcaaggctgaaccgcatcgtgtatctagtaggtttgaaaatattcacaatggaccaggggtagtgcaatctatacaccacatg
 331 Q E F K A E P H R V S S R F E N I H N G P G V V Q S I H H M
1081 cttcagtggtacctagttgaggcagcttcatatttacacaagcttcatttaaagtgtatgaatatgtttataatgactgcatttgatatg
 361 L Q W Y L V E A A S Y L H K L H L K C M N M F I M T A F D M
1171 caaagagacattctgatcactccaaagaggatagagtttgcaaggaagagagaggcagaactttatgactccttaaaaggattagcagaa
 391 Q R D I L I T P K R I E F A R K R E A E L Y D S L K G L A E
1261 gagaggcaggtccagatcagtgtgatgatacgagagacggtggaaaacatggaggacgaattggtagaagaggctgtgaagtgtgacctt
 421 E R Q V Q I S V M I R E T V E N M E D E L V E E A V K C D L
1351 agagatattgaagaaagtcaagaggtggaagggatttcccagagtaaagccgtcaaacaatgcacacattgtattaaggaattagtattg
 451 R D I E E S Q E V E G I S Q S K A V K Q C T H C I K E L V L
1441 accagactccgggctgcagtggtggaacgcctggtaagctctgtggaccacctccaggacagctaccttgggaccctagagagatgcttg
 481 T R L R A A V V E R L V S S V D H L Q D S Y L G T L E R C L
1531 cagagtttagagacagaagaggataaggagtcatcagcgagtcatgctcttcaaaagattctgaattcagcctatcaagttgaggtcagt
 511 Q S L E T E E D K E S S A S H A L Q K I L N S A Y Q V E V S
1621 gtgcgatccagctcatctgttgtgaaggtgttctttgaaagaatgaaggaaattctgcagtctatgaaacctttcaaggcaccacctgct
 541 V R S S S S V V K V F F E R M K E I L Q S M K P F K A P P A
1711 cttaatgaagaatggaagcagaaggttgctcgcaccatgattcacaaccttgacgatcacaaacttgccaagagtatttgttctcagttc
 571 L N E E W K Q K V A R T M I H N L D D H K L A K S I C S Q F
1801 agaagtcgtcttcaaaactcccatgattcattccttgcatccttgcatcagctagaagctaaacactcaggccgtttggagaaaactgaa
 601 R S R L Q N S H D S F L A S L H Q L E A K H S G R L E K T E
1891 gagaagagaatgaaggtccgaaagcaccatgcacccgtactggccaggctggcccttgatagcacatctttcaaagataaagtcctacat
 631 E K R M K V R K H H A P V L A R L A L D S T S F K D K V L H
1981 ggaatgccaaagcttgaacgtgaaataggtagagggcagtatggcgtagtgtattcctgcaaggcctggggtagcctcacacagtgtgct
 661 G M P K L E R E I G R G Q Y G V V Y S C K A W G S L T Q C A
2071 gttaaatctgtggtaccacctgatgacaaacattggaatgacttggccatggaattccattacacaaggtcaattccacctcacaagaga
 691 V K S V V P P D D K H W N D L A M E F H Y T R S I P P H K R
2161 attgttgccattgctgggtcagtagttgatcagggttatggaggaggtggctgctccctggcagtacttcttattatggagagaatgcaa
 721 I V A I A G S V V D Q G Y G G G G C S L A V L L I M E R M Q
2251 agagatttgcattcaggaatcaagatgggtcttgatctgcctagtcgactgcaaatagccctagacgtagtggaagggatccgttacttg
 751 R D L H S G I K M G L D L P S R L Q I A L D V V E G I R Y L
2341 cacagcttgggacttgttcacagagatataaaacttaagaatgttttgctggatgcggcaaacagaggaaaaataactgaccttggcttt
 781 H S L G L V H R D I K L K N V L L D A A N R G K I T D L G F
2431 tgcaagccagaagctatgatgagtggcagtattgttggaactccaattcatatggctccagagttatttactggcaagtatgacaacagt
 811 C K P E A M M S G S I V G T P I H M A P E L F T G K Y D N S
2521 gtggatacctatgcatttggaattctcttctggtacattatagctggtcatgtcaagcttccccagaactttgagcagtgtcacaacaaa
 841 V D T Y A F G I L F W Y I I A G H V K L P Q N F E Q C H N K
2611 gaccacttgtggtcttccgtcaagaaaggtaccagaccagagagactgaggcattttgatgatgaatgttgggatctaatggtgagctgt
 871 D H L W S S V K K G T R P E R L R H F D D E C W D L M V S C
2701 tgggcaggagaaactacacatcgccctctacttggtgtagtgcatgagaggcttgaagctttgcactaccaagctttagcaagaattgca
 901 W A G E T T H R P L L G V V H E R L E A L H Y Q A L A R I A
2791 ccaaaagtgaccattcctgatatacagacagacccatcatgctaccaatga
 931 P K V T I P D I Q T D P S C Y Q *
H. leucospilota MLKL
 1 atggatatgatgaaggtgggtgcccgtggagacttcgctatctgttcagggagatgctaccctacccctgtatctcacacacccagtgca
 1 M D M M K V G A R G D F A I C S G R C Y P T P V S H T P S A
 91 gctagggtattacattttgatcgcacgataaaggaaaatatgcacagcctgctgatagatataaacgaagttttcttaagaaccaaaatg
 31 A R V L H F D R T I K E N M H S L L I D I N E V F L R T K M
 181 ttcttcgaaaagcttgaaaagtatgaaagtgacacagggattgtgatgttagtcaagagggttgtgaagtactacgagattgaacacaaa
 61 F F E K L E K Y E S D T G I V M L V K R V V K Y Y E I E H K
 271 tttaaggactttaatcggatgttggttgattttgaagatgacttacaagtaaaacaaaaggatattggctttgaagaggctatagatact
 91 F K D F N R M L V D F E D D L Q V K Q K D I G F E E A I D T
 361 catttagtcgttcatactttgaggcaggagcaacagaaccgtgtcgctgacttcgagaattttaagaactttgtgatggaaagattcaat
 121 H L V V H T L R Q E Q Q N R V A D F E N F K N F V M E R F N
 451 gaagaaaaggaacacagggaatttgtggaggagcatatgaggaagttgcagttttatgaaagacgcaatgaaaaacctttaagtgaggga
 151 E E K E H R E F V E E H M R K L Q F Y E R R N E K P L S E G
 541 aattattggaccatacacactgctgtccttgacaaggagaaggtgacagtgaaaagaatcagagatgtaaatgaccataatgtacaggca
 181 N Y W T I H T A V L D K E K V T V K R I R D V N D H N V Q A
 631 tcatttcaaaaagaaactgtgaatttgatgagattttctcgaggaagtattgttcgtgtaattggaatgtgtgatgaggaacatgagaag
 211 S F Q K E T V N L M R F S R G S I V R V I G M C D E E H E K
 721 tttcttgtccttgagtacatggagaagggtgatctcagtacccttcttcataactctggagagaaaatcagccttagcaacaaagttacc
 241 F L V L E Y M E K G D L S T L L H N S G E K I S L S N K V T
 811 ctagcaacaactgctgcaagggcattctatgtcatgagccacaaattggtccatacatctttgagaacagagaagtttcttgttgataag
 271 L A T T A A R A F Y V M S H K L V H T S L R T E K F L V D K
 901 tactataccgctaagctttcaggtatgagatatgcaaaaacattttcctctgccaggagatatggtagcaaaaagccagtcgtcgatctt
 301 Y Y T A K L S G M R Y A K T F S S A R R Y G S K K P V V D L
 991 acgtgctaccttgcgcctgaactataccagccaagagagctctccccagaagttgatgtatatgggtttggaattgtcctttgggaaatc
 331 T C Y L A P E L Y Q P R E L S P E V D V Y G F G I V L W E I
1081 ttcacccaaacaaagccattttcaacattggccaaggagataggcaaagagccaactcatgcagaagtgaagaagtttgttgtacacgat
 361 F T Q T K P F S T L A K E I G K E P T H A E V K K F V V H D
1171 aaaggggaggagtacgatgttggcagtggtcccatagagaccatggtgggtgaaattatcagagactgcagggcattggacccaaagaaa
 391 K G E E Y D V G S G P I E T M V G E I I R D C R A L D P K K
1261 cgaccaagtgtgtcagaaattctgaatagactggaaaaggttgggaacgaactagaagacgaagaagatggctgtcagtag
 421 R P S V S E I L N R L E K V G N E L E D E E D G C Q *
Pkinase / PK_Tyr_Ser-Thr
ANK
COR
WD40
LRR
ROC
C
Figure S1. Nucleotide and deduced amino acid sequence of sea cucumber H. leucospilota RIPK5, RIPK7, and MLKL. Positions of nucleotide and amino acid sequences are indicated on lift sides. The different colored amino acid sequences indicate the corresponding functional domains.
