# Supplementary material for: Genomic and Transcriptional Analysis of the Necroptosis Pathway Elements RIPK and MLKL in Sea Cucumber, Holothuria leucospilota
Source: Genes (Basel). 2024 Oct 3;15(10):1297. doi: 10.3390/genes15101297 (PMC11507063; doi:10.3390/genes15101297)
Supplement: Supplementary file 1 [file genes-15-01297-s001.zip › Supplement Tables .pptx]

## Slide 1
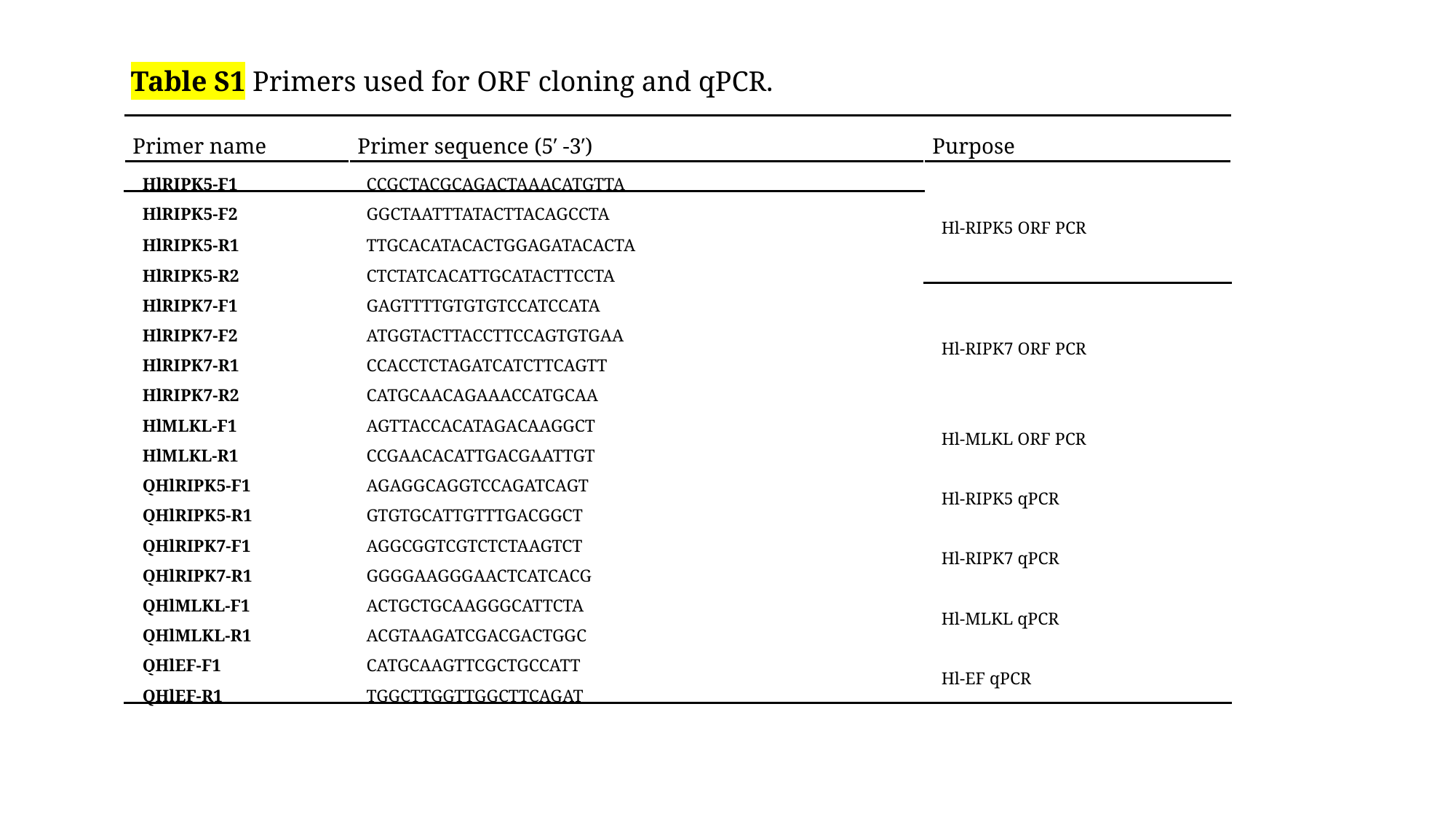

Table S1 Primers used for ORF cloning and qPCR.
| Primer name | Primer sequence (5′ -3′) | Purpose |
| --- | --- | --- |
| HlRIPK5-F1 | CCGCTACGCAGACTAAACATGTTA | Hl-RIPK5 ORF PCR |
| HlRIPK5-F2 | GGCTAATTTATACTTACAGCCTA | |
| HlRIPK5-R1 | TTGCACATACACTGGAGATACACTA | |
| HlRIPK5-R2 | CTCTATCACATTGCATACTTCCTA | |
| HlRIPK7-F1 | GAGTTTTGTGTGTCCATCCATA | Hl-RIPK7 ORF PCR |
| HlRIPK7-F2 | ATGGTACTTACCTTCCAGTGTGAA | |
| HlRIPK7-R1 | CCACCTCTAGATCATCTTCAGTT | |
| HlRIPK7-R2 | CATGCAACAGAAACCATGCAA | |
| HlMLKL-F1 | AGTTACCACATAGACAAGGCT | Hl-MLKL ORF PCR |
| HlMLKL-R1 | CCGAACACATTGACGAATTGT | |
| QHlRIPK5-F1 | AGAGGCAGGTCCAGATCAGT | Hl-RIPK5 qPCR |
| QHlRIPK5-R1 | GTGTGCATTGTTTGACGGCT | |
| QHlRIPK7-F1 | AGGCGGTCGTCTCTAAGTCT | Hl-RIPK7 qPCR |
| QHlRIPK7-R1 | GGGGAAGGGAACTCATCACG | |
| QHlMLKL-F1 | ACTGCTGCAAGGGCATTCTA | Hl-MLKL qPCR |
| QHlMLKL-R1 | ACGTAAGATCGACGACTGGC | |
| QHlEF-F1 | CATGCAAGTTCGCTGCCATT | Hl-EF qPCR |
| QHlEF-R1 | TGGCTTGGTTGGCTTCAGAT | |

## Slide 2
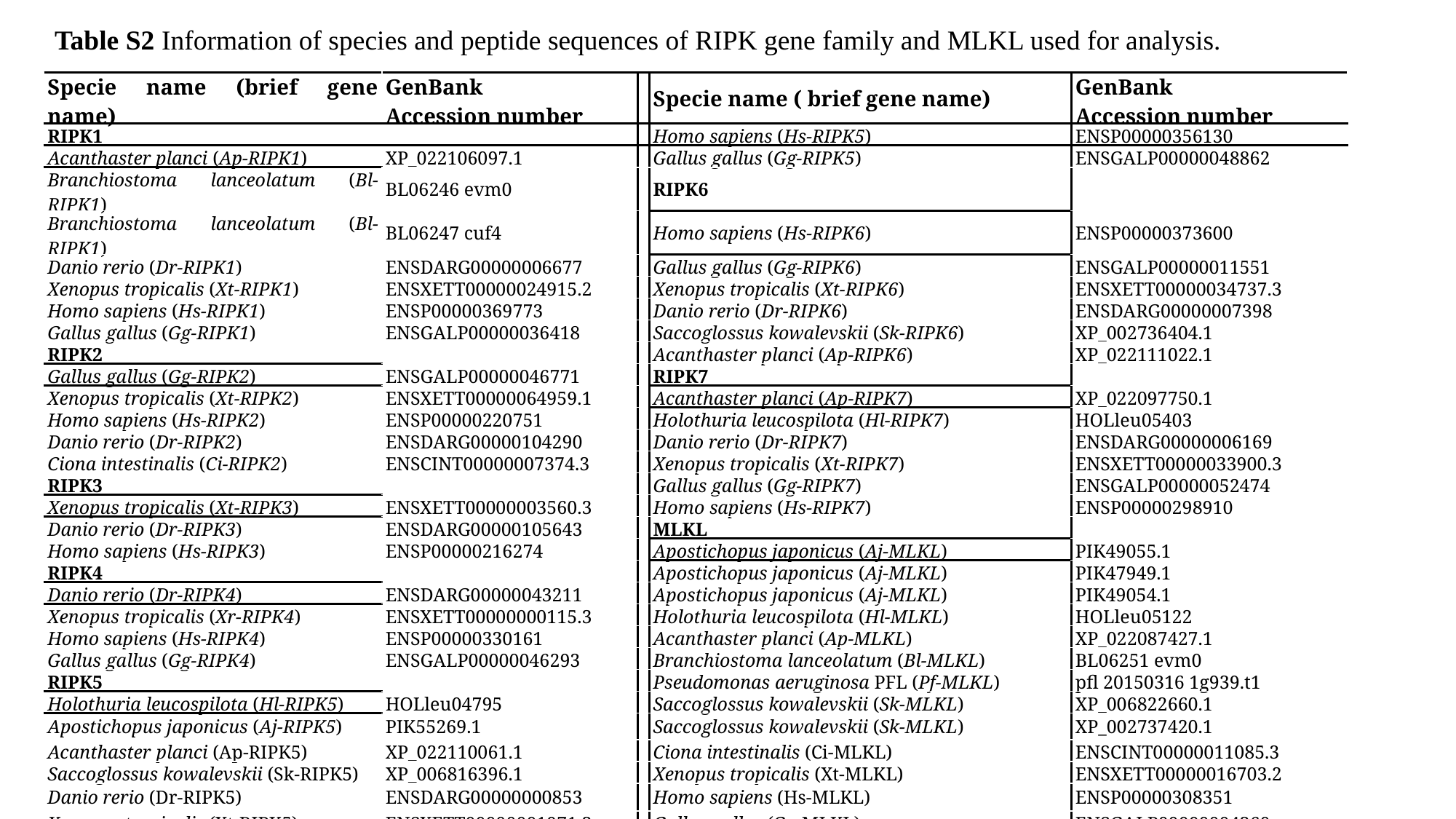

Table S2 Information of species and peptide sequences of RIPK gene family and MLKL used for analysis.
| Specie name (brief gene name) | GenBank Accession number | | Specie name ( brief gene name) | GenBank Accession number |
| --- | --- | --- | --- | --- |
| RIPK1 | | | Homo sapiens (Hs-RIPK5) | ENSP00000356130 |
| Acanthaster planci (Ap-RIPK1) | XP\_022106097.1 | | Gallus gallus (Gg-RIPK5) | ENSGALP00000048862 |
| Branchiostoma lanceolatum (Bl-RIPK1) | BL06246 evm0 | | RIPK6 | |
| Branchiostoma lanceolatum (Bl-RIPK1) | BL06247 cuf4 | | Homo sapiens (Hs-RIPK6) | ENSP00000373600 |
| Danio rerio (Dr-RIPK1) | ENSDARG00000006677 | | Gallus gallus (Gg-RIPK6) | ENSGALP00000011551 |
| Xenopus tropicalis (Xt-RIPK1) | ENSXETT00000024915.2 | | Xenopus tropicalis (Xt-RIPK6) | ENSXETT00000034737.3 |
| Homo sapiens (Hs-RIPK1) | ENSP00000369773 | | Danio rerio (Dr-RIPK6) | ENSDARG00000007398 |
| Gallus gallus (Gg-RIPK1) | ENSGALP00000036418 | | Saccoglossus kowalevskii (Sk-RIPK6) | XP\_002736404.1 |
| RIPK2 | | | Acanthaster planci (Ap-RIPK6) | XP\_022111022.1 |
| Gallus gallus (Gg-RIPK2) | ENSGALP00000046771 | | RIPK7 | |
| Xenopus tropicalis (Xt-RIPK2) | ENSXETT00000064959.1 | | Acanthaster planci (Ap-RIPK7) | XP\_022097750.1 |
| Homo sapiens (Hs-RIPK2) | ENSP00000220751 | | Holothuria leucospilota (Hl-RIPK7) | HOLleu05403 |
| Danio rerio (Dr-RIPK2) | ENSDARG00000104290 | | Danio rerio (Dr-RIPK7) | ENSDARG00000006169 |
| Ciona intestinalis (Ci-RIPK2) | ENSCINT00000007374.3 | | Xenopus tropicalis (Xt-RIPK7) | ENSXETT00000033900.3 |
| RIPK3 | | | Gallus gallus (Gg-RIPK7) | ENSGALP00000052474 |
| Xenopus tropicalis (Xt-RIPK3) | ENSXETT00000003560.3 | | Homo sapiens (Hs-RIPK7) | ENSP00000298910 |
| Danio rerio (Dr-RIPK3) | ENSDARG00000105643 | | MLKL | |
| Homo sapiens (Hs-RIPK3) | ENSP00000216274 | | Apostichopus japonicus (Aj-MLKL) | PIK49055.1 |
| RIPK4 | | | Apostichopus japonicus (Aj-MLKL) | PIK47949.1 |
| Danio rerio (Dr-RIPK4) | ENSDARG00000043211 | | Apostichopus japonicus (Aj-MLKL) | PIK49054.1 |
| Xenopus tropicalis (Xr-RIPK4) | ENSXETT00000000115.3 | | Holothuria leucospilota (Hl-MLKL) | HOLleu05122 |
| Homo sapiens (Hs-RIPK4) | ENSP00000330161 | | Acanthaster planci (Ap-MLKL) | XP\_022087427.1 |
| Gallus gallus (Gg-RIPK4) | ENSGALP00000046293 | | Branchiostoma lanceolatum (Bl-MLKL) | BL06251 evm0 |
| RIPK5 | | | Pseudomonas aeruginosa PFL (Pf-MLKL) | pfl 20150316 1g939.t1 |
| Holothuria leucospilota (Hl-RIPK5) | HOLleu04795 | | Saccoglossus kowalevskii (Sk-MLKL) | XP\_006822660.1 |
| Apostichopus japonicus (Aj-RIPK5) | PIK55269.1 | | Saccoglossus kowalevskii (Sk-MLKL) | XP\_002737420.1 |
| Acanthaster planci (Ap-RIPK5) | XP\_022110061.1 | | Ciona intestinalis (Ci-MLKL) | ENSCINT00000011085.3 |
| Saccoglossus kowalevskii (Sk-RIPK5) | XP\_006816396.1 | | Xenopus tropicalis (Xt-MLKL) | ENSXETT00000016703.2 |
| Danio rerio (Dr-RIPK5) | ENSDARG00000000853 | | Homo sapiens (Hs-MLKL) | ENSP00000308351 |
| Xenopus tropicalis (Xt-RIPK5) | ENSXETT00000001971.3 | | Gallus gallus (Gg-MLKL) | ENSGALP00000004360 |
| | | |
| --- | --- | --- |
